# AI-enabled ChatGPT or LLM: a new algorithm is required for plagiarism-free scientific writing

**DOI:** 10.1097/JS9.0000000000000939

**Published:** 2023-11-23

**Authors:** Soumen Pal, Manojit Bhattacharya, Md. Aminul Islam, Chiranjib Chakraborty

**Affiliations:** a School of Mechanical Engineering, Vellore Institute of Technology, Vellore, Tamil Nadu, India; b Department of Zoology, Fakir Mohan University, Vyasa Vihar, Balasore, Odisha, India; c Department of Biotechnology, School of Life Science and Biotechnology, Adamas University, Kolkata, West Bengal, India; d Advanced Molecular Lab, Department of Microbiology, President Abdul Hamid Medical College, Karimganj, Kishoreganj; e COVID-19 Diagnostic Lab, Department of Microbiology, Noakhali Science and Technology University, Noakhali, Bangladesh

*Dear Editor*,

A recent article by Shafiee demonstrated the use of ChatGPT for scientific writing, especially the writing of a research paper^1^. Several other researchers have also informed ChatGPT’s capability for scientific writing and argued about the same^2,3^. Therefore, Shafiee’s article in this journal is very important and timely.

Plagiarism is a significant concern within academic and scientific writing. Plagiarism and errors may have dire consequences for the credibility of researchers, and it creates adverse effects on the undertaken research. Therefore, academicians, researchers, and scientists always avoid plagiarism in scientific writing. The advent of artificial intelligence (AI)-generated content has only exacerbated the issue by facilitating the ease with which individuals can copy and paste information. Kleebayoon and Wiwanitkit^4^ examined the ethical ramifications of AI technologies that generate text which evades plagiarism detectors. This group reported that while generative AI chatbots may not possess the capability to produce wholly referenced text, they have the potential to create blog posts or student essays. Therefore, it threatens the credibility of evaluations and scholarly work. AI-generated output harbors inaccuracies, which can prove problematic in scientific and medical contexts. For example, ChatGPT, a human-like conversational chatbot developed by OpenAI in November 2022, was examined for its medical report generation capabilities. It was discovered that ChatGPT’s responses had errors, and researchers have highlighted these^5^. ChatGPT may struggle to generate precise information in areas with little existing research. If AI-generated content is perceived as less trustworthy due to issues related to plagiarism or errors, it may be less likely to be understood as accurate. In academic and scientific contexts, the presence of plagiarism and errors in AI-generated content has a considerable impact on the credibility of information. Interestingly, several anti-plagiarism services have been developed in academic writings, namely Turnitin., iThenticate, PlagiarismDetect, etc. However, it is crucial to acknowledge that AI-generated content can also be used for cheating purposes, and the current plagiarism detection methods may not possess the necessary efficacy to detect all instances of plagiarism. Since ChatGPT sources its content from pre-existing online content, inadvertent similarities with the original content may be present. Most plagiarism detection scores, when utilizing ChatGPT-generated content, have a considerable amount of plagiarism. Therefore, it is of utmost need that while utilizing ChatGPT, the generated content remains original.

The present algorithms utilized by ChatGPT operate on the generative pre-trained transformer (GPT) format of a large language model (LLM), demonstrating its competence in various natural language processing tasks without necessitating adaptation on downstream data^6^. LLM uses the transformer model, which uses deep learning-based architecture. This model works faster compared to the previous neural network model. Numerous academic datasets have been used to assess ChatGPT’s effectiveness in various tasks, and the results reveal that it is proficient in achieving different objectives^7^. However, the current algorithms utilized to tackle the issues of plagiarism and errors in AI-generated content are not entirely immune to limitations. In the specific context of ChatGPT, apprehensions have been expressed regarding the potential for bias and plagiarism when utilizing the tool^8^. ChatGPT may reproduce and reinforce pre-existing biases in the data on which it is trained, resulting in flawed and inequitable predictions.

Moreover, there exists a danger of intellectual theft if individuals directly replicate and paste data created by ChatGPT without offering appropriate citation or recognition of its usage. Despite efforts to produce solutions for recognizing factual inaccuracies in summarization models, the continuously changing nature of summarization systems, metrics, and annotated benchmarks leads to factual evaluation becoming a shifting concept, thus hindering clear-cut comparisons among its measures^9^. Furthermore, most recent algorithms in factual detection have been made on synopses from older (pre-transformer) models rather than more current summarization models. Designing a new algorithm for ChatGPT, which generates output that is free of error and plagiarism, demands a fusion of diverse techniques and approaches. To achieve this objective, some steps must be followed. Before initiating algorithmic training, it is crucial to preprocess and sanitize the data. This phase entails eliminating irrelevant information, such as stop words, and rectifying spelling and grammatical inaccuracies. The textual data must undergo scrutiny for plagiarism detection using dependable tools. The algorithm can be trained by employing machine learning and deep learning techniques. One such approach is utilizing the Naive Bayes classification algorithm, which can categorize text into distinct groupings, including positive or negative sentiment^10^. Another technique is to utilize natural language generation methods, which involve creating a natural language text generation system capable of generating human-readable languages such as English and Chinese through AI and linguistic methodologies^11^. To optimize the precision of the algorithm, calibration before deployment is imperative. The calibration process necessitates the adjustment of the output probabilities of the algorithm to guarantee the uniformity of the predictions across all responses^12^. To ensure that the output is devoid of any discrepancies and plagiarism, the utilization of a verifier module is indispensable. This module can be trained to function as a verifier for ChatGPT’s output, thereby enhancing its performance iteratively by utilizing fine-grained corrective instructions^13^. For evaluating the algorithm’s performance, appropriate evaluation metrics need to be employed. One of the viable approaches for this end is the adoption of exact match as the evaluation metric, which entails comparing the model’s produced output with the ground-truth data.

In conclusion, a new plagiarism-free algorithm is urgently needed to generate plagiarism-free text for scientific writing. Developing a new plagiarism-free and error-free algorithm for ChatGPT provides numerous benefits. Although ChatGPT can generate responses that resemble those of humans, incomplete or biased data may result in errors. By eliminating errors and plagiarism, a new algorithm can enhance the quality and reliability of ChatGPT’s output. The implications of relying on current versions of ChatGPT for high-stakes decision-making can be severe, but a new algorithm can ensure that the output is error-free and plagiarism-free, thereby enhancing user trust. Although ChatGPT has been successfully implemented for writing in education, healthcare, and other domains, a new algorithm can expand its applicability in these areas. For instance, in education, ChatGPT can generate plagiarism-free and high-quality essays and research papers; while in healthcare, it can produce accurate medical reports, treatment suggestions, and plagiarism-free high-quality other documents. Therefore, all professionals in education, healthcare, and other domains should come together with computer engineers and computer scientists and get involved immediately in developing the next-generation algorithm, which will help students, teachers, researchers, and scientists for plagiarism-free scientific writing.

## Ethical approval

Not applicable.

## Sources of funding

Not applicable.

## Author contribution

S.P.: writing – review and editing, investigation, and validation; M.B. and M.A.I.: validation; C.C.: conceptualization, data curation, writing – original draft, and review and editing. All authors critically reviewed and approved the final version of the manuscript.

## Conflicts of interest disclosure

All authors report no conflicts of interest relevant to this article.

## Research registration unique identifying number (UIN)

1. Name of the registry: not applicable.
2. Unique identifying number or registration ID: not applicable.
3. Hyperlink to your specific registration (must be publicly accessible and will be checked): not applicable.

## Guarantor

Md. Aminul Islam, COVID-19 Diagnostic Lab, Department of Microbiology, Noakhali Science and Technology University, Noakhali 3814, Bangladesh; E-mail: aminulmbg@gmail.com

## Data availability statement

The data in this correspondence article are not sensitive in nature and are accessible in the public domain. The data are therefore available and not of a confidential nature.

## Provenance and peer review

Not commissioned, internally peer-reviewed.

## References

[1] Shafiee A . Matters arising: authors of research papers must cautiously use ChatGPT for scientific writing. Int J Surg 2023;109:2853–2854. 37222674 10.1097/JS9.0000000000000515 PMC10498857

[2] Baker N Thompson B Fox D . ChatGPT can write a paper in an hour - but there are downsides. Nature 2023. doi:10.1038/d41586-023-02298-x. [Epub ahead of print]. 37438635

[3] Salvagno M Taccone FS Gerli AG . Can artificial intelligence help for scientific writing? Crit Care 2023;27:75. 36841840 10.1186/s13054-023-04380-2 PMC9960412

[4] Kleebayoon A Wiwanitkit V . Artificial intelligence, chatbots, plagiarism and basic honesty: comment. Cell Mol Bioeng 2023;16:173–174. 37096073 10.1007/s12195-023-00759-x PMC10121937

[5] Metze K Morandin-Reis RC Lorand-Metze I . The amount of errors in ChatGPT’s responses is indirectly correlated with the number of publications related to the topic under investigation. Ann Biomed Eng 2023;51:1360–1361. 37061596 10.1007/s10439-023-03205-1

[6] Qin C Zhang A Zhang Z . Is ChatGPT a general-purpose natural language processing task solver? arXiv preprint arXiv 2023;2302:06476.

[7] Laskar MTR Bari MS Rahman M . A systematic study and comprehensive evaluation of ChatGPT on benchmark datasets. arXiv preprint arXiv 2023;2305:18486.

[8] Sánchez-Ruiz LM Moll-López S Nuñez-Pérez A . ChatGPT challenges blended learning methodologies in engineering education: a case study in mathematics. Appl Sci 2023;13:6039.

[9] Tang L Goyal T Fabbri AR . Understanding factual errors in summarization: errors, summarizers, datasets, error detectors. arXiv preprint arXiv 2022;2205:12854.

[10] Erfina A Nurul MR . Implementation of Naive Bayes classification algorithm for Twitter user sentiment analysis on ChatGPT using Python programming language. Data Metadata 2023;2:45.

[11] Huang Y and Yu H . Research on text generation techniques combining machine learning and deep learning. In: Proceedings of the Third Asia-Pacific Conference on Image Processing, Electronics and Computers, 2022:319–26.

[12] Zhao Z Wallace E Feng S, . Calibrate before use: improving few-shot performance of language models. In: Proceedings of the 38th International Conference on Machine Learning, PMLR 139, 2021:12697–12706.

[13] Han J Collier N Buntine W . PiVe: prompting with iterative verification improving graph-based generative capability of LLMs. arXiv preprint arXiv 2023;2305:12392.

